# Retraction note: Anti-inflammatory and protective investigations on the effects of Theranekron® “an alcoholic extract of the Tarantula cubensis” on wound healing of peritoneal in the rat: an in vivo comparative study

**DOI:** 10.1186/s13000-016-0575-2

**Published:** 2016-11-02

**Authors:** Farajollah Adib-Hashemi, Farshad Farahmand, Shamim Fattah Hesari, Bijan Rezakhaniha, Ehsan Fallah, Amir Farshid Fayyaz, Masoomeh Dadpay

**Affiliations:** 1 Department of Clinical Science, Faculty of Veterinary Medicine, Tehran University, Tehran, Iran; 2 Graduated, Faculty of Veterinary Medicine, Garmsar Branch, Islamic Azad University, Garmsar, Iran; 3 Department of Surgery, Orthopedic Surgeon, Imam Reza Hospital, Tehran, Iran; 4 Department of Urology, Imam Reza Hospital, Tehran, Iran; 5 Department of Orthopedic and Trauma Surgery, AJA University of Medical Sciences, Tehran, Iran; 6 Departmen of Legal Medicine, AJA University of Medical Sciences, Tehran, Iran; 7 Department of Pathology, Imam Reza Hospital, AJA University of Medical Sciences, Tehran, Iran

The Editor-in-Chief and Publisher have retracted this article [1] because the scientific integrity of the content cannot be guaranteed. An investigation by the Publisher found it to be one of a group of articles we have identified as showing evidence suggestive of attempts to subvert the peer review and publication system to inappropriately obtain or allocate authorship. This article showed evidence of plagiarism (most notably from the articles cited [2–5]) and peer review and authorship manipulation.
